# Pooled prevalence and genetic diversity of norovirus in Africa: a systematic review and meta-analysis

**DOI:** 10.1186/s12985-022-01835-w

**Published:** 2022-06-28

**Authors:** Dessie Tegegne Afework, Mulu Kebede Shumie, Getachew Ferede Endalew, Aschalew Gelaw Adugna, Baye Gelaw Tarekegn

**Affiliations:** 1 Department of Medical Laboratory Sciences, College of Health Sciences, Debre Tabor University, P.O. Box 272, Debre Tabor, Ethiopia; 2 Department of Medical Microbiology, College of Medicine and Health Sciences, University of Gondar, P.O. Box 196, Gondar, Ethiopia; 3 Department of Nursing, College of Health Sciences, Debre Tabor University, P.O. Box 272, Debre Tabor, Ethiopia

**Keywords:** Norovirus, Gastroenteritis, Prevalence, Systematic review, Meta-analysis, Genogroups, Genotypes, Africa

## Abstract

**Background:**

Noroviruses are the leading cause of acute gastroenteritis in all age groups globally. The problem is magnified in developing countries including Africa. These viruses are highly prevalent with high genetic diversity and fast evolution rates. With this dynamicity, there are no recent review in the past five years in Africa. Therefore, this review and meta-analysis aimed to assess the prevalence and genetic diversity of noroviruses in Africa and tried to address the change in the prevalence and genetic diverisity the virus has been observed in Africa and in the world.

**Methods:**

Twenty-one studies for the pooled prevalence, and 11 out of the 21 studies for genetic characterization of norovirus were included. Studies conducted since 2006, among symptomatic cases of all age groups in Africa, conducted with any study design, used molecular diagnostic methods and reported since 2015, were included and considered for the main meta-analysis. PubMed, Cochrane Library, and Google Scholar were searched to obtain the studies. The quality the studies was assessed using the JBI assessment tool. Data from studies reporting both asymptomatic and symptomatic cases, that did not meet the inclusion criteria were reviewed and included as discussion points. Data was entered to excel and imported to STATA 2011 to compute the prevalence and genetic diversity. Heterogeneity was checked using I^2^ test statistics followed by subgroup and sensitivity analysis. Publication bias was assessed using a funnel plot and eggers test that was followed by trim and fill analysis.

**Result:**

The pooled prevalence of norovirus was 20.2% (95% CI: 15.91, 24.4). The highest (36.3%) prevalence was reported in Ghana. Genogroup II noroviruses were dominant and reported as 89.5% (95% CI: 87.8, 96). The highest and lowest prevalence of this genogroup were reported in Ethiopia (98.3%), and in Burkina Faso (72.4%), respectively. Diversified genotypes had been identified with an overall prevalence of GII. 4 NoV (50.8%) which was followed by GII.6, GII.17, GI.3 and GII.2 with a pooled prevalence of 7.7, 5.1, 4.6, and 4.2%, respectively.

**Conclusion:**

The overall pooled prevalence of norovirus was high in Africa with the dominance of genogroup II and GII.4 genotype. This prevalence is comparable with some reviews done in the same time frame around the world. However, in Africa, an in increasing trained of pooled prevalence had been reported through time. Likewise, a variable distribution of non-GII.4 norovirus genotypes were reported as compared to those studies done in the world of the same time frame, and those previous reviews done in Africa. Therefore, continuous surveillance is required in Africa to support future interventions and vaccine programs.

**Supplementary Information:**

The online version contains supplementary material available at 10.1186/s12985-022-01835-w.

## Introduction

Acute gastroenteritis (AGE) is the second greatest burden of all infectious diseases worldwide, in which the burden is high in low-income countries [1]. Rotavirus and norovirus (NoV) are the leading causes of AGE globally [2]. In Africa, NoV outbreaks and associated gastroenteritis were first reported in South Africa in 1993 with the two strains Norwalk (GI.1) and Hawaii (GII.1) [3].

Noroviruses are a group of non-enveloped viruses having a single-stranded positive-sense RNA genome. They are assigned to the *Caliciviridae *family [4]. Based on the variation on the major capsid protein (VP1) coding region, NoVs are classified into 10 genogroups (GI-GX) and 49 genotypes. From these genogroups, GI, GII, and GIV are known to infect humans [5]. Genogroup II is by far the most frequently reported genogroup comprising about 27 genotypes. According to some previous studies, GII.4 is the predominant genotype responsible for about 55–97% of the disease burden [6, 7] with an increased evolution rate due to accumulated mutations in the capsid region [8]. As a result of this, different GII.4 variant strains including the GII.4 US95/96 strain in 1995, GII.4 Farmington Hills in 2002, GII.4 Hunter in 2004, GII.4 Den Haag in 2006, GII.4 New Orleans in 2009, and GII.4 Sydney in 2012 [9] were reported. A new variant, GII.4 Hong Kong, was also reported recently in China that are associated with increased outbreaks [10]. Non-GII.4 NoVs commonly GII.17, GII.3, GII.6, GII.10, and GII.2 were also reported in developing countries including Africa [11–13]

Nowadays NoVs are considered as the leading cause of diarrheal death over 5 years of age, and the second in under 5 children with similar patterns across the globe [2]. It had been also reported as the leading cause of food-borne associated gastroenteritis [14]. As a result of this, it is responsible for about 10–20% of hospitalizations, severe diarrhea, dehydration, and death in the elderly and under 5 children [15].

In developing countries, NoV-associated mortality had been previously estimated to be as high as 212,000 deaths per year in under 5 children [16]. According to one review in developing countries, the prevalence of NoV was 17% [17]. Another epidemiological review of NoV in African children with gastroenteritis reported an overall prevalence of 13.5% [13]. Similarly, a systematic review of studies performed between 1993 and June 2015 among children in Sub-Saharan Africa also revealed a prevalence of 12.6% [18].

Increased awareness of this diversified pathogen has placed considerable interest in developing diagnostics, antivirals, and vaccines, as well as finding more effective ways to interrupt its transmission. Although primary studies had been performed in various countries in Africa, and one systematic review was reported in 2016, organized and up-to-date systematic review and meta-analysis has not been investigated recently in Africa. Some primary studies showed an increased prevalence of NoV with high genetic diversity in Africa [11, 19–21], and in other areas too [10, 22]. In this vaccine development era with an increased emergence of new genetic variants, updated and organized data is a priority which provides a space for the present study. Therefore, the purpose of this systematic review and meta-analysis is to assess the pooled prevalence and genetic diversity of NoVs in Africa.

## Review questions

The research questions were developed based on the condition, context, and population (CoCoPop) approach. This systematic review and meta-analysis involve two review questions:

1. Has the pooled prevalence of NoVs among individuals with gastroenteritis in Africa changed in the past 5 years, and is it consistent with global prevalence?
2. Are the genogroup and genotype distribution of NoVs in Africa highly variable and differ from previous studies?

## Methods and materials

### Study area and period

A total of 21 studies reporting their finding between 2015 and 2021 all from the five regions of Africa (six studies each in Central and East Africa; four in South Africa, two studies from each North and West African country) were considered. The review process was conducted from March 01, 2021, to May 30, 2021. From the 21 studies, 9,238 individual participants were considered for the evaluation of the pooled prevalence of NoV. On the other hand, 11 of the 21 studies which reported genogroup and genotypic information were used for the assessment of the genetic diversity of NoV in Africa.

### Review protocol development

Cochrane Library, Google Scholar, PubMed, and other databases were checked for the presence of similar studies to avoid duplication. To answer the review questions, a review protocol was developed and registered on PROSPERO with the registration number CRD42021251475.

### Search strategy

Cochrane Library, PubMed, and Google Scholar were searched to identify potential articles on the prevalence and genetic diversity of NoVs in Africa. The following terms with Medical Subject Headings (MeSH) and all fields were used to search. Boolean operators (AND, OR, NOT) were also used to narrow and widen the scope of our search. Publications were identified using the search terms “epidemiology”, “molecular epidemiology” “prevalence”, “burden”, “norovirus”, “human norovirus infection”, “human caliciviruses” “acute gastroenteritis”, “acute diarrhea”, “Africa” and related terms.

The studies were assessed repeatedly in three consecutive steps to determine their eligibility. In step 1, the studies were evaluated considering only the information presented in the title and abstract. When the abstract was available, the study was further assessed for full texts. The full texts of the articles selected for step 2 were read to evaluate their eligibility and adequacy for data extraction; in step 3 the studies were re-evaluated to determine their eligibility for meta-analysis and systematic reviews of the outcome variables. Search results were combined into the EndNote X9 file and duplicates were removed.

### Eligibility criteria

*Inclusion criteria *In this meta-analysis, studies that reported the prevalence and genetic diversity of NoVs in all ages of human subjects in African countries. Studies carried out in hospitalized patients, outpatient departments and community settings were included. In addition, studies that employed cross-sectional, case control and national surveillances, molecular technique and those published in English language were included. Studies that recruited with at least 50 study participants were considered eligible. Furthermore, studies were also considered for this analysis irrespective of the duration of the study.

*Exclusion criteria *Review articles, meta-analysis and those studies that did not satisfy the required information indicated in the inclusion criteria were excluded. As most studies did not consider seasonality, it could not be established for norovirus infections in this review too.

### Data extraction

Two independent reviewers made an abstract and full-text review. When there was a disagreement, a discussion was made to reach a consensus with the involvement of a 3rd reviewer. From the eligible articles, data were extracted and sorted by the following variables: the leading author, reporting year and study period, country, study design, African region, age group, diagnostic methods used, number of positives, genotyped samples, and genotypes.

### Quality appraisal

This systematic review and meta-analysis was performed following the Preferred Reporting Items for Systematic Reviews and Meta-Analyses (PRISMA) guideline [23]. The Joanna Briggs Institute (JBI) quality appraisal checklist was used to assess the quality of individual papers [24].

### Data analysis

The retrieved data was entered to excel and then imported to STATA version 11. Primarily the fixed effect model was done. However, a random-effect model was employed at the end due to the existence of heterogeneity between studies. The estimated proportion of each genogroup (GI and GII NoVs) was assessed by dividing the number of each genogroup by the total genotyped samples. The overall pooled prevalence and genotype distribution was calculated and displayed on graphs and tables. Heterogenicity was assessed by using the forest plot and Galbraith plot. Publication bias was checked by inspecting the funnel plot symmetry (subjectively) and by Egger's tests (objectively), with a significant publication bias at (*p* > 0.05) [25]. Trim and Fill analysis was used to see the effect of publication bias.

## Result

### Characteristics of included studies

**Fig. 1:**
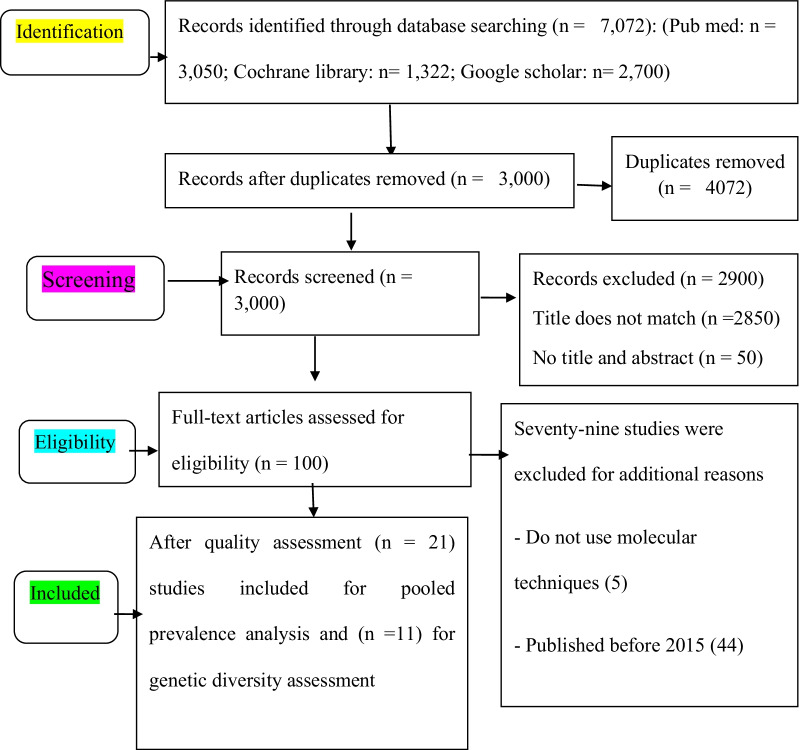
PRISMA study selection flow diagram of included studies for analysis

A total of 7072 research articles had been retrieved from all the databases. After removing duplications 3000 articles were left for further analysis. In the second step, 2900 articles were excluded after reading their titles and abstracts and the remaining 100 studies were assessed for full-text review. Seventy-nine studies were excluded for additional reasons. Finally, twenty-one of the articles were considered for the assessment of the overall prevalence of NoV. Eleven out of the 21 studies articles were considered for the genetic diversity study (Fig. 1).

In this review, NoV prevalence was defined as a proportion of NoV positive cases to the total number of gastroenteritis cases screened. The proportions of studies based on data from outpatients, communities and hospitalized patients were 8/21 (38%) each, 8/21 (38%), and 5/21 (23.8%), respectively. More generally, 13/21 (61.9%) studies were conducted in the health facility as compared to 8/21 (38.1%) studies conducted in the community setting. Similarly, most of the studies 16/21 (76.2%) were conducted among under 5 children while the remaining 5/21 studies were conducted in older ages. Most of the included articles were from Central and East African regions. Similarly, most of the studies (57.14%) were cross-sectional studies. All the included studies employed molecular diagnostic methods and were reported between 2015 to 2021. Furthermore, almost all of the included studies were conducted since 2010, except one study conducted in Kenya between 2006 to 2009.

### The pooled prevalence of norovirus in Africa

**Fig. 2:**
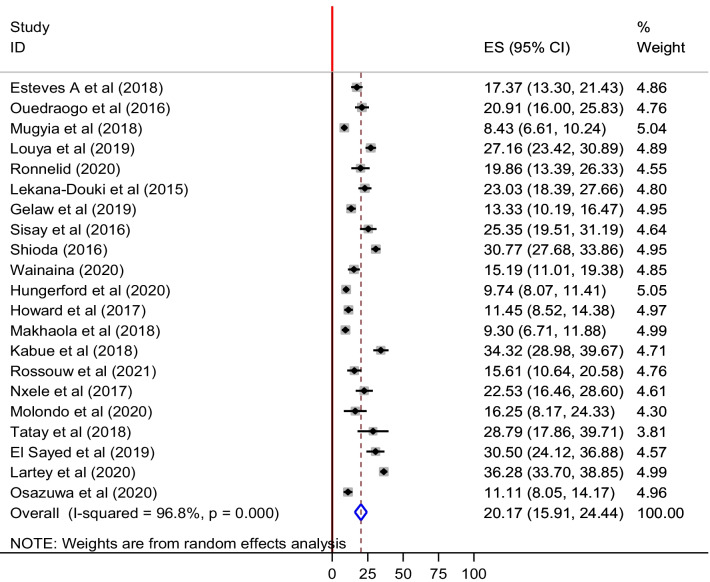
Forest plot of pooled prevalence of NoV among individuals with gastroenteritis in Africa: The pooled prevalence represented by the X-axis, and the list of included papers represented by Y-axis, The red line represents the minimum possible prevalence value (0). The dashed line represents the mean pooled NoV prevalence estimate. The gray box represents the weight of each study contributing to the pooled prevalence estimate. The black dot at the center of the gray box represents the point prevalence estimate of each study and the horizontal line indicates the 95% confidence interval for estimates of each study. The blue diamond represents the 95% confidence interval of the pooled NoV prevalence estimate

A total of 9,238 stool samples, from 21 studies, had been analyzed for the assessment of the pooled prevalence of NoV (Table 1). According to the subjective assessment of the Galbraith plot (Additional file 1: Figure S1), we had observed heterogenicity among individual studies. Therefore, we applied a random-effect model for estimating the overall prevalence. Accordingly, the overall pooled prevalence of NoV was 20. 2% (95% CI: 15.9, 24.4). The highest (36.3%), and lowest prevalence (8.4%), were reported in Ghana and Cameroon, respectively. As shown in the forest plot (Fig. 2), statistically significant heterogeneity was identified (I^2^ = 96.8%; *p*-value < 0.001). As there was significant heterogeneity among the studies subgroup and sensitivity analysis was done.

**Table 1:** Descriptive summary of 21 studies included in the meta-analysis for the pooled prevalence of human noroviruses in Africa among studies reported from 2015 to 2021

Author	Study period	Country	African region	Type of study	Age	Diagnosis method	Sample size	Total positive	Samples genotyped	Ref
Esteves et al. (2018)	June 2012–Oct. 2013	Angola	Central Africa	Cross-sectional	< 5	Rt-qPCR	334	58	46	[52]
Ouedraogo et al. (2016)	Nov. 2011-Sept. 2012	Burkinafaso	Central Africa	Case control	< 5	Rt-qPCR	263	55	29	[19]
Mugyia et al. (2019)	Jan. 2010–Dec. 2013	Cameroon	Central Africa	Surveillance	< 5	RT-PCR	902	76	76	[42]
Louya et al. (2019)	June 2012–June 2013	Congo	Central Africa	Surveillance	< 5	RT-qPCR	545	148	None	[53]
Ronnelid et al. (2020)	Jan.–Dec. 2015	Burkinafasso	Central Africa	Cross-sectional	< 5	Rt-qPCR	146	29	24	[54]
Lekana-D et al. (2015)	March 2010–June 2011	Gabon	Central Africa	Surveillance	< 5	Multiplex PCR	317	73	None	[55]
Gelaw et al. (2019)	Nov. 2015–April 2016	Ethiopia	East Africa	Cross-sectional	< 5	Rt-qPCR	450	60	60	[11]
Sisay et al. (2016)	June–Sept. 2013	Ethiopia	East Africa	Cross-sectional	All ages	RT-PCR	213	54	22	[12]
Shioda et al. (2016)	Oct. 2006–Feb. 2009 & June 2007–Oct. 2008	Kenya	East Africa	Surveillance	All ages	RT-PCR	858	264	None	[56]
Wainaina et al. (2020)	April–June 2017	Kenya	East Africa	Cross-sectional	Adult	RT-PCR	283	43	None	[57]
Hungerford et al. (2020)	Nov. 2012–Dec. 2015	Malawi	East Africa	Case control	< 5	Rt-qPCR	1211	118	64	[58]
Howard et al. (2017)	July 2012–Oct. 2013	Zambia	East Africa	Surveillance	< 5	RT-PCR	454	52	None	[59]
Makhaola et al. (2018)	July 2013–Dec. 2015	Botswana	South Africa	Cross-sectional	< 5	Rt-qPCR	484	45	33	[60]
Kabue et al. (2018)	July 2014– Aril 2015	South Africa	South Africa	Cross-sectional	< 5	Rt-qPCR	303	104	None	[61]
Rossouw et al. (2021)	July 2013–Dec. 2017	South Africa	South Africa	Cohort	< 5	Multiplex PCR	205	32	28	[26]
Nxele et al. (2017)	June–August 2014	South Africa	South Africa	Cross-sectional	< 5	Rt-qPCR	182	41	None	[62]
Molondo et al. (2020)	August 2017–Oct. 2018	South Africa	South Africa	Cross-sectional	< 5	Rt-qPCR	80	13	4	[27]
Tatay et al. (2018)	June–May 2017	Sudan	North Africa	Cross-sectional	< 5	Rt-qPCR	66	19	None	[63]
Elsayed et al. (2019)	Jan. 2018–May 2019	Egypt	North Africa	Cross-sectional	< 18	Rt-qPCR	200	61	None	[64]
Lartey et al. (2020)	Jan. 2008–Dec. 2017	Ghana	West Africa	Surveillance	< 5	Rt-qPCR	1337	485	None	[65]
Osazuwa et al. (2020)	March 2018–Feub. 2019	Nigeria	West Africa	Case control	< 5	Rt-qPCR	405	45	45	[21]

In our review, three out of 21 studies reported the prevalence of NoV among asymptomatic individuals. The average prevalence of NoV in these studies was 7.1% (95% CI: 5.2, 9.4). More specifically, a study conducted in Burkina Faso reported the prevalence of NoV as 6% in asymptomatic individuals where as to 20.9% among symptomatic cases. Similarly, a study in Malawi reported a prevalence of 8.54%, and 12.15% for asymptomatic and symptomatic cases, respectively. However, a zero prevalence of NoV was reported among asymptomatic controls, and 11.1% among symptomatic cases in a study conducted in Nigeria.

### Subgroup analysis

The studies were stratified by study area (African region), study design, sample size, age group, and molecular diagnostic methods used to identify the possible source of heterogeneity. Based on this analysis, the pooled prevalence of norovirus among under 5 children was 19.25 (95%CI: 14.4, 23.5), whereas the prevalence of NoV among studies participants older than 5 years of age was 23.77% (95% CI: 15.9, 33.5). The pooled prevalence of NoV in community-based, hospitalized and outpatient studies were 25.24, 20.30, and 18.68%, respectively (Table 2).

**Table 2:** Subgroup analysis for the pooled estimate of NoVs from 21 studies reported from 2015 to 2021

Sub groups	Number of studies included	Pooled estimate of NoVs in % (95% CI)	Heterogeneity: I^2^ (*p*-value)
*African region*			
Central Africa	Six	19.4 (12.0, 26.7)	95.5% (< 0.001)
East Africa	Six	17.5 (10.9, 24.4)	96.8% (< 0.001)
South Africa	Five	19.5 (10, 29)	94.7% (< 0.001)
North Africa	Two	30 (24.6, 35.6)	0.0% (0.79)
West Africa	Two	25.8 (23.9, 27.8)	99.3% (< 0.001)
*Age group*			
Under5 children	Sixteen	19.25 (14.4, 23.5)	96.8% (< 0.001)
Other age groups	Five	23.77 (15.9, 33.5)	91.8% (< 0.001)
*Diagnosis method*			
Rt-qPCR	Fourteen	21 (15.4, 26.8)	97% (< 0.001)
Conventional RT-PCR	Five	18 (9, 27)	97.6% (< 0.001)
Multiplex PCR	Two	19.37 (12.1, 26.6)	78.2% (0.032)
*Study design*			
Cross-sectional	Eleven	20.7 (15.4, 18.2)	91.2% (< 0.001)
Case control	Three	13.4 (8.2, 18.6)	98.8% (< 0.001)
Surveillance	Six	22.8 (12.3, 33.4)	98.8 (< 0.001)
*Sample size*			
< 300	Nine	21.2 (17.6, 24.8)	97% (< 0.001)
> 300–500	Seven	16.8 (11.7, 21.9)	68.9% (< 0.001)
> 500	Five	22.4 (11, 33.9)	99.2% (< 0.001)

### Sensitivity analysis

Sensitivity analysis revealed no significant difference except for some outlier studies that deviate from the overall estimate. As all the studies lie within the 95% confidence interval the pooled prevalence is not affected by the individual study (Additional file 1: Figure S2).

### Genetic diversity of noroviruses

**Fig. 3:**
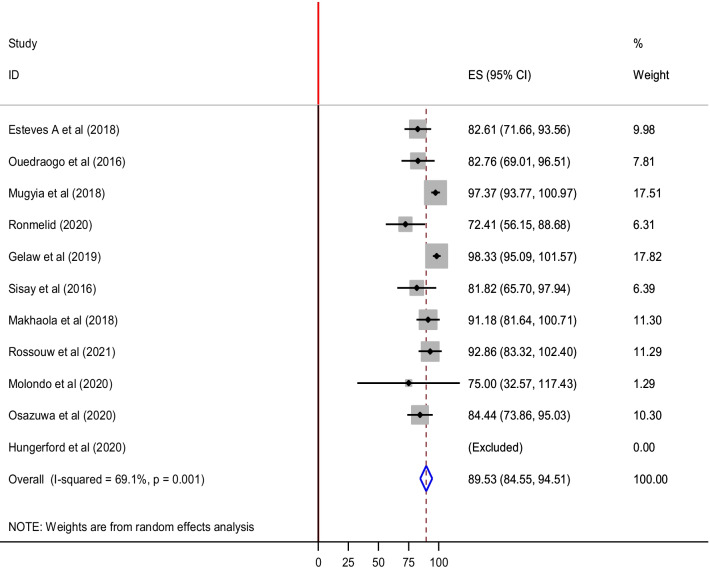
GII NoVs among all eleven molecularly characterized samples: The pooled prevalence GII NoVs had been represented by the X-axis, and the list of included papers represented by Y-axis, The bold vertical line represents the minimum possible prevalence value (0). The dashed line represents the mean pooled GII NoV prevalence estimate. The gray box represents the weight of each study contributing to the pooled prevalence estimate. The black dot at the center of the gray box represents the point prevalence estimate of each study and the horizontal line indicates the 95% confidence interval for estimates of each study. The blue diamond represents the 95% confidence interval of the pooled GII NoV prevalence estimate

Eleven out of the 21 studies that were conducted between 2010 and 2019 were used to assess the genetic diversity of NoV. Almost all of the studies had reported both GI and GII NoVs except one study conducted in Malawi that reported only GII NoV. The prevalence of GII was by far higher in all of the studies. The pooled prevalence of GII NoV was 89.5% (95% CI: 84.6, 94.5%) with a substantial heterogeneity (I^2^ = 69.1%; *p*-value = 0.001). The highest pooled prevalence of GII NoV had been reported as 98.3, and 97.4% in Ethiopia, and Cammeroon, respectively while the lowest was reported in in Burkina Faso (72.4%) (Fig. 3).

In this review, the dominance GII.4 NoV genotypes was also observed. This was followed by GII.6, GII.17, and GII.2, respectively. Similarly, GI.3 was the dominant genotype among all GI NoVs. The highest prevalence of non-GII.4 NoVs (GII.6) was reported in Ethiopia followed by a study conducted in Angola. The dominance of GII.17, among all non-GII.4 norovirus was also reported in the two studies conducted in Ethiopia. Similarly, the dominance of GII.7 and GII.10, from all non-GII.4 NoVs identified, was also seen in the studies done from Angola and Ethiopia, respectively. The highest prevalence of GI NoVs was reported in a study conducted in Angola. Most the included articles used open reading frame-2 (ORF-2) for genotyping and or sequencing, but some studies [12, 19, 20, 26, 27] used dual systems of genotyping using both ORF-1 and ORF-2.

**Fig. 4:**
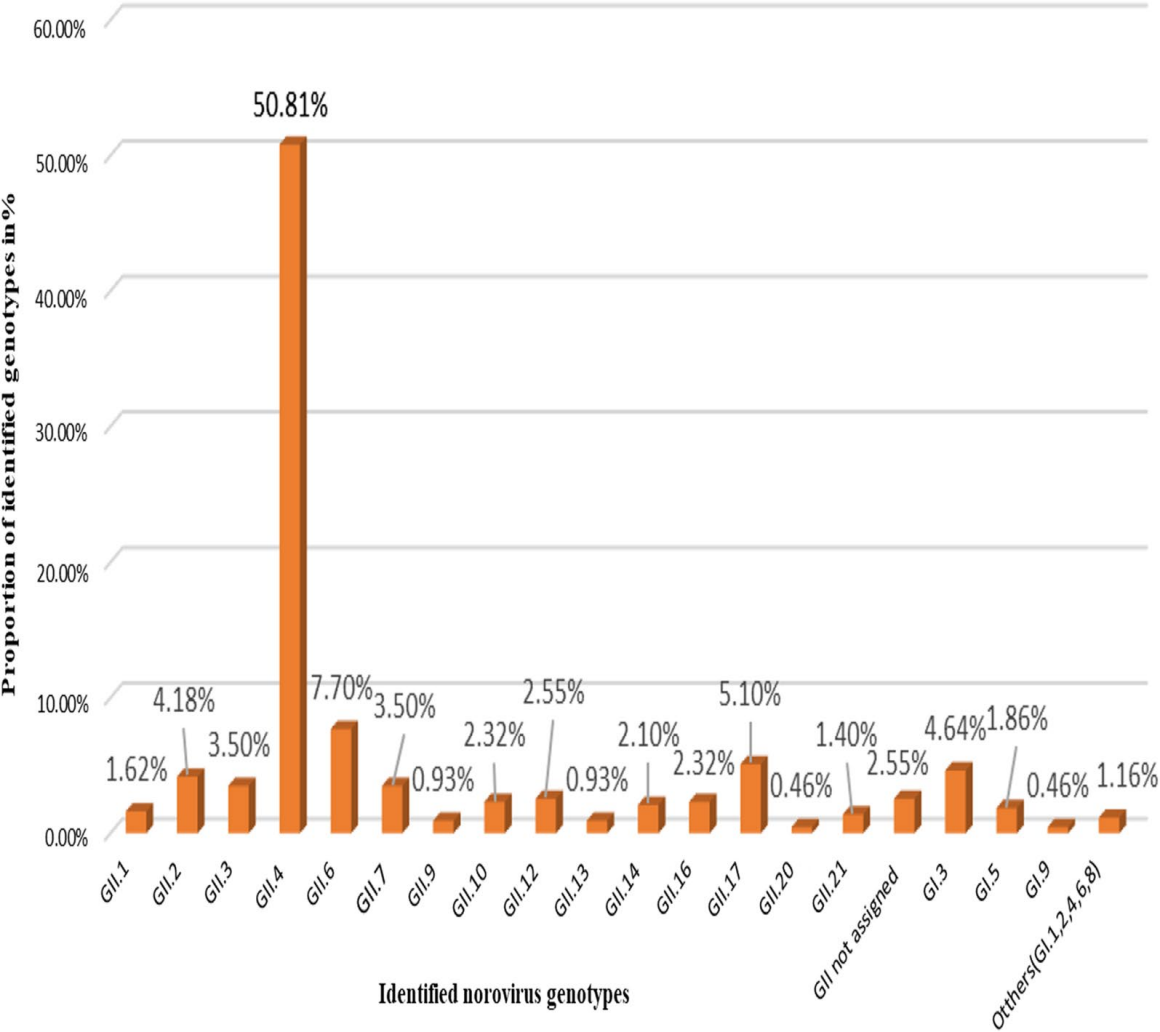
The distribution of NoV genotypes in Africa: The GII.4 is the leading among all the eleven molecularly characterized samples in our review which is followed by GII.6, GII.17. GI.3, GII.2, and others

The GII.4 was the leading genotype of all the GII NoVs (53.5%) (Additional file 1: Figure S3), and among all the reported genotypes (50.81%). However, the prevalence of non-GII.4 NoV genotypes was high with a prevalence of 7.7, 5.1, 4.6, and 4.2%, for GII. 6, GII.17, GI.3, and GII.2, respectively. Previously rare genotypes including GII.20, GII.9, GI.1, GI.2, and GI.8. were also reported in our review (Fig. 4). Recombinant types like GII.Pe/GII.4, GII. P4/GII.4 (in Botswana), GII.g/GII.12 (in Ethiopia), and GII.Pg/GII.1 (in South Africa) were also reported. The most common reported GII.4 variants in this review were Sydney 2012 and New Orleans 2009.

### Assessment of publication bias

**Fig. 5:**
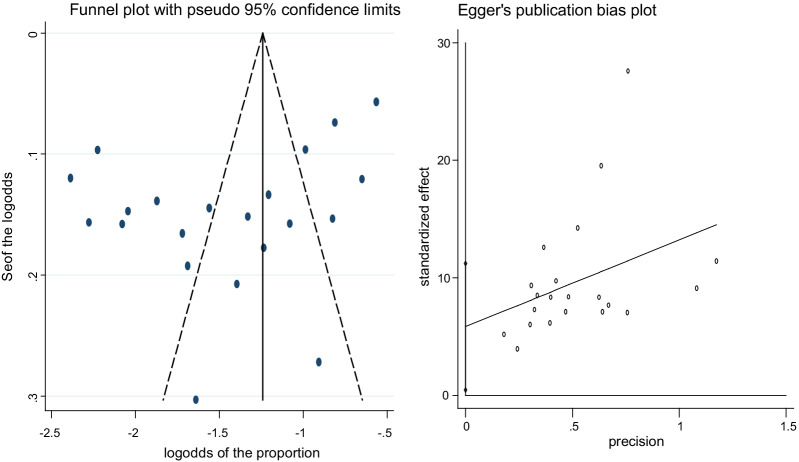
Funnel plot symmetry to check the presence or absence of publication bias: *left* The pooled prevalence of NoVs in Africa from studies reporting between 2015 to 2021; *right* Egger’s publication bias plot. Each dot represents individual studies. The x-axis represents precision (reciprocal of the standard error of the estimate). The y-axis represents log transformed standardized effect (estimate divided by its standard error)

The result of the funnel plot showed some publication bias as indicated by the slight asymmetrical distribution of articles (Fig. 5 left). Eggers tests were conducted and also confirmed the presence of publication bias (Fig. 5 right). Therefore, trim and fill analysis was done to show the effect and magnitude of the publication bias (Additional file 1: Figure S4). Similar to the pooled prevalence, an asymmetrical distribution for GII NoVs among genotyped samples were observed (Additional file 1: Figure S5 left). This asymmetry was also confirmed by Egger's tests. The results of Egger's tests showed that there was a slight statistically significant publication bias in estimating the pooled proportion of GII NoV among those genotyped samples (y-intercept = 2.45; *p*-value < 0.05) (Additional file 1: Figure S5 right).

## Discussion

Norovirus is the leading cause of non-bacterial epidemic AGE in all age groups globally [14]. The burden is high in developing countries with poor socio-economic status [1]. Several studies reported that NoVs have been frequently identified around the globe [28–32]. With a vaccine in a pipeline [33], an updated systematic review is a priority to provide comprehensive data. Therefore, this study aimed to assess the overall pooled prevalence and genetic diversity of NoV in Africa, and assure weather there is a changing prevalence and or genetic diversity of this pathogen is occured.

In this study, the pooled prevalence and genetic diversity of NoVs were reviewed and analyzed from studies conducted on patients with gastroenteritis and published between January 2015 and April 2021. According to this systematic review and meta-analysis, the pooled prevalence of NoV, among the included 21 studies, was 20.2% (95% CI: 15.9, 24.4). This study indicated the burden of NoV infection in Africa was high that might have an impact on the routine management of AGE cases. The present pooled prevalence was in agreement with one recent global review which was conducted almost in same time frame (2015 to 2020) from 45 countries across the world that was 17.7% (95% CI: 16.3%-19.2%) [34]. Our finding was also somewhat in agreement with a review done from 52 studies in China which was 16.68% (95% CI 16.63–16.72) [35].

In contrast, our finding was higher than a previous review conducted in Africa 13.5% (95% CI 12.7–14.3) [13], another review conducted from 1990 to 2013 in Africa 11% (95% CI 8–14%) [36], and a review done in Sub-Saharan Africa 14.2% published before 2015 [18]. The source of this variation could be the difference in the study participants, the study period, and the study setting. These reviews considered those studies published before 2015. This might be associated with an increased prevalence and dynamic change or evolution of the virus from time to time, and the domination of this virus over the others [5, 22]. The other source of variation might also be the exclusion criteria sated as they excluded outbreak cases, and included only sporadic cases in their analysis [36].

More specifically, the pooled prevalence of NoV among under 5 children was lower among under 5 children 19.25 (95%CI: 14.4, 23.5) as compared to the prevalence of NoV among studies participants older than 5 years of age 23.77% (95% CI: 15.9, 33.5). This was in agreement with the recent global report among under 5 children 19.0 (95% CI: 17.3–20.8) and older than 5 years 17.6% (95% CI: 13.0% –23.5%) [34]. A similar agreement of prevalence had been reported in one study done in China with children ≤ 5 years 17.39% (95% CI: 17.32–17.47), and among adult patients 19.28% (95% CI: 19.23–19.33) [35]. The pooled prevalence was also higher in community-based studies that is 25.24% (95% CI: 20.2, 28.9) as compared to 20.30, and 18.68% for studies conducted among hospitalized, and OPD, respectively. A comparable gradient of decreasing prevalence was also reported from one previous global systematic review conducted from 1997 to 2021 that is 20, 18, and 15% for studies conducted in community, outpatients, and hospital settings, respectively [37]. A comparable result was also reported in another previous global systematic review with 24, 20, and 17% for community and outpatient settings, and inpatient settings, respectively [14].

In our review, three studies reported the prevalence of NoV among asymptomatic individuals with an average prevalence of 7.1% (95% CI 6.3–9.1). This was in agreement with the prevalence of NoV in asymptomatic patients reported from 28 studies across the globe done in the same time frame with our study that was 6.7% (95% CI: 5.1%-8.8%) [34]. Our finding was, however, slightly lower than a previous report in Africa that reported an overall prevalence of 9.7% (95% CI 8.4–11.1) among asymptomatic groups [13], and a review in Sub-Saharan Africa children was (9.2%) [6]. The difference might be due to the time frame when the review was conducted. That is, we included studies published 2015 and 2021 while other previous African studies were considered those studies published before 2015. The other discrepancy might be the number of included studies reporting asymptomatic participants. For example, for the one reporting 9.2% from a review in done Sub-Saharan Africa, the number of included studies were 8 as compared to our review that only included 3 studies.

Genogroup GII is the leading NoV genogroup in the world [34, 38]. In this study, from all the included eleven studies for genetic analysis, the commonest NoV genogroup detected was NoV GII which was 89.5% (95% CI 84.5,94.5) with a small proportion of NoV GI (8.4%). Our finding was in agreement with the recent global review done almost in a similar time frame that reported as 92.9% (95% CI: 90.6%–94.6%) for GII, and 6.7% (95% CI: 5.2%–8.5%) for GI [34]. The same is true for the previous review conducted in Africa where GII strains represented 88.5% of all detected NoVs, and GI strains 3.4% [13]. In contrast to our finding, a lower prevalence of GII (76.4%), with an increased prevalence of GI NoVs (21.7%) was reported in the previous review done in Sub-Saharan countries [6]. The same is true for the previous review done in Africa from studies published between 1990 and 2013 that is 81% (95% CI 73–87%) for GII and, 18% (95% CI 12–24%) for GI. This might be the inclusion of studies published before 2015 and after 2015 as seen in our case that could be associated to the genetic variations because of mutation or evolution of the virus, and or recombination events through time [8, 39]. The source of discrepancy might also be due the inclusion criteria in this study. They only included age groups ≤ 17 years as compared to our review considered all age groups [40]. The other source of variation might be the setting as they only include Sub-Saharan Africa as compared to our study considered all African studies.

The GII.4 NoV genotype have been dominating in the past two decades, and hence it is responsible for a great disease burden causing outbreaks and sporadic cases of gastroenteritis [41, 42]. This genotype has high mutation rate where the new variants emerged every 2 to 3 years [22, 40]. However, the emergence of different strains of GII.4 and non-GII.4 genotypes have become a common occurrence in the past five years across different countries in the world [5, 11, 12, 22, 43]. This might be due to genetic variations because of mutation and recombination events occurred in this specific virus [8, 39].

In this review, a great diversity of NoV genotypes were reported ranging from GII.1 to GII.21, except GII.5, GII.8, GII.11, GII.15, GII.18, and GII.19, with the predominance of GII.4, 50.8% (95% CI 40.60, 66.48) from all identified genotypes (Fig. 4). This was also true for GI NoV that comprise of GI.1 to GI.9, with the dominance of GI.3 (4.6%) of all the reported NoV genotypes. The great diversity and distribution of different genotypes in our finding was in agreement with the previous review in Sub-Saharan Africa except for some differences in the number of genotype distributions [18]. Our finding was in agreement with the previous studies done in Africa that also reported the dominance of GII.4 NoVs which was 54.1% [13], 65.2% for a review in Sub-Saharan Africa [6]. A similar finding was observed in the recent global studies with 59.3% (95% CI: 53.4%–64.9%) [34], and 52% [38]. These findings assured the dominance of GII.4 NoVs in the world generally and in Africa specifically.

Next to GII.4, GII.6, GII.17, GI.3and GII.2 with a prevalence of 7.7%, 5.1%, 4.6%, 4.2%, respectively were also highly reported in our review. This report is in agreement with a previous study where an increased prevalence of non-GII.4 NoV had been reported in different parts of China and Japan as a single variant or as a recombinant type [44–46]. These might be associated with an increased prevalence of other genotypes through time other than the GII.4 NoV which include GII.17, GII.6, GII.2, and GII.3 in different countries since 2014 with the most prevalent genotype change occurred during the 2015–2017 period which can increase cross transmission from one setting to another through time [11, 12, 45, 47–49]. In contrast to this the previous review conducted in Africa by 2016 reported a dominance of other genotypes including GII.3 (12.2%), GI.3 (3.6%), and GII.6 (3%), with a lower prevalence rate of GII.17, and GII.2 was reported. A similar dominance of GII.3 (14%), that was followed by GII.2 (11%), and GII.6 (5%) genotypes was also reported in one global review [38]. The same is true for another global review done from 2015 to 2020 reported a high prevalence of (14.9%, 95% CI: 10.6%–20.5%) for GII.3 [34]. An increased prevalence of GII.2 (50%), and GII.17 (15.38%) between 2014 to 2019 were also reported in China [35]. This showed the highest evolutionary dynamics of NoV strains from time to time that demonstrates a changing prevalence and diversity of NoV genotypes over time in Africa and globally [14, 50, 51]

Among all genotyped NoVs, GI.3 with 4.6% was the dominant genotype with in GI NoVs that was followed by GI.5 (1.9%). This was in agreement with other reviews, where a dominance of GI.3 NoV was reported in the previous review done in Africa 3.5% [13], and in a recent review done in the world 4% [38].

Our finding reported a substantial to considerably high heterogenicity as measured in the subjective and objective assessment methods. Therefore, we stratified studies into the study region, diagnostic method, study design, severity of the disease, setting, and age groups. Regional estimates of NoV infection in the subgroup analysis showed a lower prevalence of 17.5% in Eastern Africa compared to a higher prevalence of 30% in the North Africa region. This regional difference can be attributable to that in Eastern Africa, 75% of study participants were all age groups compared to the studies conducted in North Africa that considered children. Most studies in the Eastern African region also used the most sensitive diagnostic method Rt-qPCR. The prevalence of NoV infection differs based on laboratory diagnostic methods used. Higher prevalence was detected by Rt-qPCR (21%) as compared to conventional RT-PCR (18%) (Table 2). In this review, the sensitivity analysis revealed no significant difference except for some outlier studies that deviate from the overall estimate. As all the studies lie within the 95% confidence interval the pooled prevalence is not affected (Additional file 1: Figure S2). On the other hand, a significant publication bias had been reported. The reason might be due inclusion of only published papers and or studies published in the English language.

## Limitations of this review

The first limitation of this study was that our finding reported a substantial to considerably high heterogenicity as measured in the subjective and objective assessment methods which might have an effect on the pooled prevalence. The source of this observed heterogeneity could be due to differences in the spatio-temporal and epidemiological patterns of the included studies. We tried to minimize this by doing subgroup analysis and sensitivity analysis. Another issue was that only English articles or reports, and published studies had been considered for analysis which might be associated with some of the observed publication bias in our report. Although the seasonality of NoV infections was important for effective health care planning, in our review we could not establish the seasonality as it was inconsistent in the individual studies. In addition to this, the review was made in 21 studies conducted from 15 out of the 54 African countries which might be difficult to generalize or conclude for Africa. Despite this limitation, our study provided updated information regarding NoV associated gastroenteritis and its genetic diversity, which is relevant for vaccine development and to implement intervention mechanisms.

## Conclusion and recommendation

The overall pooled prevalence of NoV in Africa was still high, with more than one-fifth of the individuals with AGE are affected. This finding might be used to point out and estimate the burden of NoV-associated AGE in Africa in the past 5 years, where there is a lack of surveillance systems. It is also important to developed diagnostic and management approaches. The pooled prevalence of NoV in our review is high and comparable with some reviews and studies done in the same time frame around the world including China. However, in Africa, as compared to those previous studies conducted in the different time frame, the pooled prevalence is in the increasing trained through time. Except for a few data, the genotype distribution of NoV in Africa was high and somewhat different from the previous global and African trends. The GII and GII.4 were the dominant genogroup and genotype, respectively. However, a relative increase in the distribution of unusual genotypes including, but not limited to GII.6, GII.17, GII.2, and others was observed in the region as compared to the dominance of other non-GII.4 genotypes that include GII.3 from those previous reviews done in Africa with a different time frame as well as GII.2, GII.3, and GII.17 in the world reported in the same time frame. In line with this, although GII genogroup and GII.4 genotypes are still dominating in the world and in Africa generally, non-GII.4 Therefore, continuous networked surveillance is a priority to provide updated data that target future interventions and inform vaccine development strategies in the continent.

## Supplementary Information

**Additional file 1**. Supplementary figures.

## Data Availability

The dataset(s) supporting the conclusions of this article is (are) included within the article and the data that support the findings of this study are available from the corresponding author [DT], upon reasonable request.

## Abbreviations

AGE: Acute gastroenteritis
GI: Genogroup I
GII: Genogroup II
JBI: Joanna Briggs Institute
MeSH: Medical subject heading
NoV: Norovirus
OPD: Outpatient department
ORF: Open reading frame
PRISMA: Preferred reporting items for systematic reviews and meta-analyses
RNA: Ribonucleic acid
RT-PCR: Reverse transcriptase polymerase chain reaction
Rt-qPCR: Real time quantitative polymerase chain reaction
STATA: Statistics and data
WHO: World Health Organization
