# Supplementary material for: Pooled prevalence and genetic diversity of norovirus in Africa: a systematic review and meta-analysis
Source: Virol J. 2022 Jun 28;19:115. doi: 10.1186/s12985-022-01835-w (PMC9238157; doi:10.1186/s12985-022-01835-w)
Supplement: Supplementary file 1 — Additional file 1. Supplementary figures. [file 12985_2022_1835_MOESM1_ESM.docx]

**Supplementary materials for the pooled prevalence of norovirus in Africa**

**Additional file 1: Figure S1** Galbraith plot for subjective assessment of heterogenicity of the pooled prevalence of NoV:

Authors’ name represents individual studies. The y-axis represents standard error of the estimates. The x-axis represents the reciprocal of the standard error. In this figure, some of the weighted estimates of individual studies represented by the authors’ name lie outside the + 2 or -2 standard error of the pooled estimate. This subjective assessment indicates the presence of heterogeneity between individual studies as well.

**Additional file 1: Figure S2** Sensitivity analysis on the pooled prevalence of NoVs in Africa:

The pooled prevalence had been represented by the X-axis, and the list of included papers assessed for sensitivity analysis had been represented by Y-axis

Almost all the individual studies are with in the 95% confidence interval limit.

**Additional file 1: Figure S3** Forest plot showing the pooled proportion of GII.4 among GII NoV positive samples in Africa:

The pooled prevalence GII.4 NoVs had been represented by the X-axis, and the list of included papers represented by Y-axis

The bold vertical line represents the minimum possible prevalence value (0). The dashed line represents the mean pooled GII.4 NoV prevalence estimate. The gray box represents the weight of each study contributing to the pooled prevalence estimate. The black dot at the center of the gray box represents the point prevalence estimate of each study and the horizontal line indicates the 95% confidence interval for estimates of each study. The blue diamond represents the 95% confidence interval of the pooled GII.4 NoV prevalence estimate.

**Additional file 1: Figure S4** A diagram dealing with publication bias applying the trim and fill analysis for the pooled prevalence of NoV to adjust the observed publication bias:

The small open dots represent the actual studies while the large dotes in the bottom represent the filled studies.

**Additional file 1: Figure S5** Funnel plot for assessing publication bias for the pooled prevalence of GII NoV among genotyped samples (the values were log transformed):

**Left**: The subjective assessment of this funnel plot looks asymmetrical which is an indication of publication bias. Each dot represents individual studies. The y-axis represents standard error of estimate. The x-axis represents log transformed estimates. **Right**: egger's test result for assessing publication bias in GII NoV among genotyped samples: The figure showed the presence of publication; the data had been log transformed.
